# “This course is like a compass to us” – a qualitative study on newly settled migrants’ perceptions of civic and health orientation in Sweden

**DOI:** 10.1186/s12889-021-11654-3

**Published:** 2021-08-31

**Authors:** Maissa Al-Adhami, Katarina Hjelm, Josefin Wångdahl, Elin C. Larsson

**Affiliations:** 1 Department of Women’s and Children’s Health/Department of Public Health and Caring Sciences, Uppsala University, Box 564, 751 22 Uppsala, Sweden; 2 Department of Public Health and Caring Sciences, Uppsala University, Box 564, 751 22 Uppsala, Sweden; 3 Department of Public Health and Caring Sciences, Uppsala University, Box 564, 752 22 Uppsala, Sweden; 4 Department of Global Public Health, and Department of Women’s and Children’s Health, Karolinska Institutet, SE-171 77 Stockholm, Sweden

**Keywords:** Newly settled migrants, Civic orientation, Health promotion, Health communication, Migration

## Abstract

**Background:**

Migrants face structural, socio-political barriers in their resettlement processes that negatively affect their health. Migration also adversely impacts resources such as social capital and health literacy that are of importance for health and integration into society. Hence, there is a need for health promotion in the early post-migration phase. In Sweden, newly settled refugee migrants who have received a residence permit are offered an Introduction programme including a civic orientation course. The program is intended to facilitate access to the labour market and promote integration. The aim of the study was to explore participants’ perceptions and experiences of a civic orientation course with added health communication.

**Methods:**

We performed six focus group discussions: two in Arabic, two in Farsi and two in Somali. The discussions were facilitated by native speaking moderators. Participants were 32 men and women recruited from civic orientation classes in the county of Stockholm. We used an interview guide with semi-structured questions. The data were analysed using a method for content analysis for focus group discussions.

**Results:**

Three main categories were identified: (1) ‘The course gives valuable information but needs adjustments’, which includes that the civic and health orientation is needed earlier, during the asylum phase, and that planning and course content need adjustments. (2) ‘The health communication inspired participants to focus on their health’, which includes that the health communication was useful and inspired uptake of healthier habits. (3) ‘Participation in the course promoted independence and self-confidence’, which includes that the course gave insights into society and values in Sweden, and promoted independence and new social contacts.

**Conclusion:**

This study adds knowledge about the users’ perspectives on the potential of civic orientation to promote the health and integration of newly settled migrants, describing ways in which civic orientation with added health communication promoted health and empowerment. However, the content and delivery of the course need adjustment to better fit the migrants’ life situations and varying pre-existing knowledge.

**Supplementary Information:**

The online version contains supplementary material available at 10.1186/s12889-021-11654-3.

## Background

Sweden is one of the countries that has received the largest number of refugee migrants in the EU/EES region [1]. Migrants face structural, socio-political barriers in their resettlement processes such as marginalisation and lack of adequate housing and employment, which affects their health, independent of prior health status [2, 3]. In Sweden, newly settled migrants, with a residence permit based on refugeeship or subsidiary protection status, partake in a national *Introduction program* [4]. There is a need and a potential to promote health of migrants in the early post-migration stage [5, 6]. One platform, reaching a large part of the newly arrived migrants, is the *Civic Orientation* (CO), which is a mandatory part of the Introduction program [4].

Health promotion, defined here as actions enabling increased control over and the improvement of one’s health [7], implemented early in the post-migration phase, could mediate ill health caused by systemic inequities and act as a buffer for stressors experienced by migrants [2, 5]. For instance, mental ill health e.g. depression and anxiety are commonly reported, and linked to poor socioeconomic conditions [8], lengthy asylum-seeking processes [9], and experiences of discrimination and isolation [2, 10]. Furthermore, unequal health outcomes between migrant groups and native population are reported beyond the early resettlement process, e.g. mental health, cardiovascular disease and reproductive health outcomes [11–13]. Similarly, resources such as social capital, i.e. trusting relationships, social support and networks [14] and health literacy i.e. abilities and resources to find, understand, assess and apply health information [15] are weaker among newly settled migrants than among native born [16, 17]. These resources impact health outcomes and health care utilisation, and the resettlement process as such as they influence participation and empowerment in general [14, 15].

Research on health promotion for newly settled migrants have shown that there is an array of public and non-profit services and programs, e.g. for mental health [18], but that access to these programs is limited by structural barriers [19, 20]. Furthermore, it has been suggested that research on migrants’ health would benefit from continuity and international comparisons [18]. Studies such as this, examining large-scale introduction activities, available across countries and seldom viewed from the migrants’ perspectives on health promotion, are therefore needed.

National Introduction programs focusing on newly settled migrants with a refugee background, also referred to as *Establishment Programs or Civic Integration Programs,* are common in European and OECD countries [21]. In Sweden, the program includes activities such as Swedish language training, CO, labour market counselling and other activities aimed at facilitating establishment on the labour market and active participation in society [4, 22]. Participation and fulfilment of activities entitles the individual to welfare benefits for a period of 2 years.

**Fig. 1:**
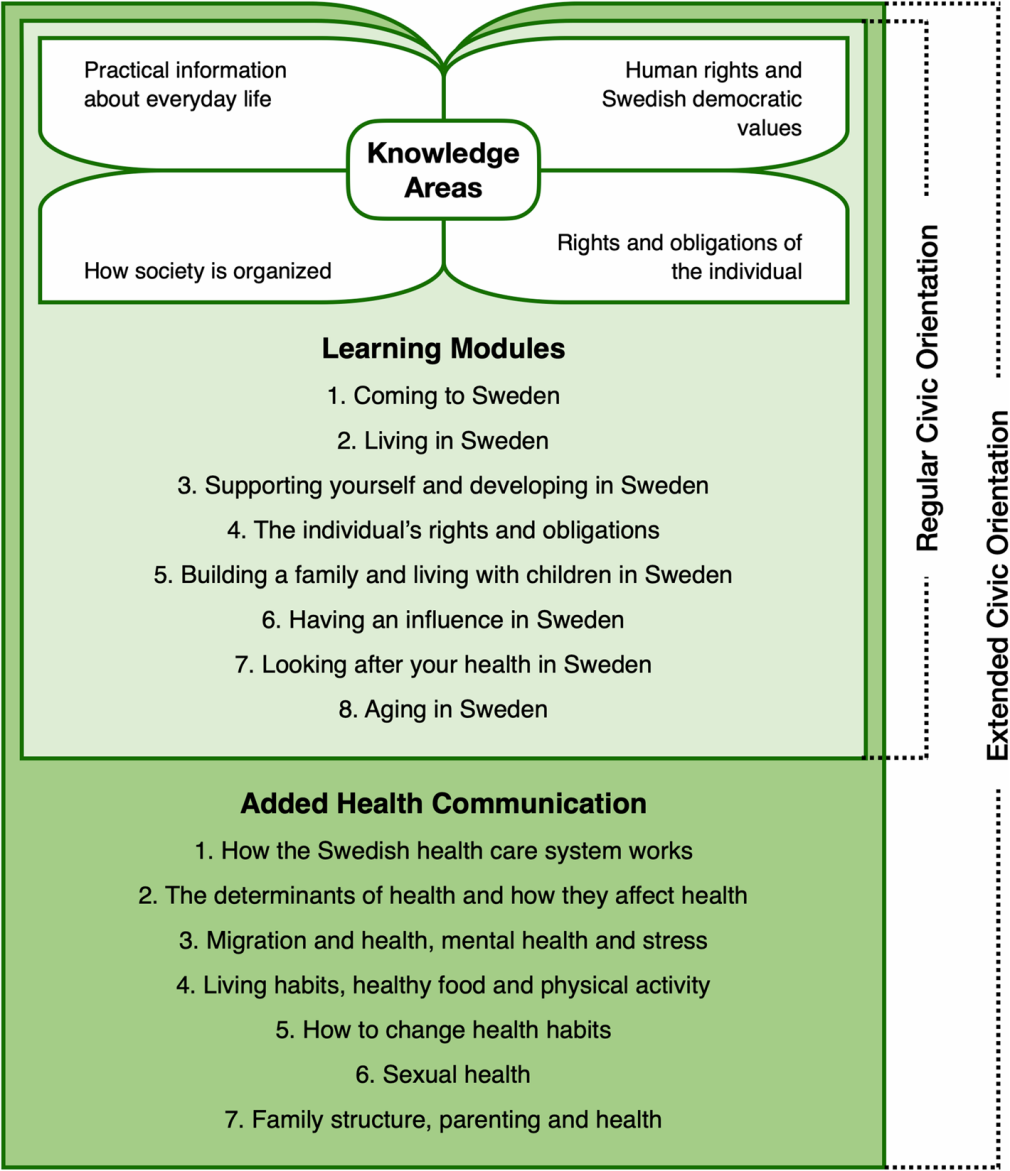
Content of regular CO course and CO extended with added health topics

The Swedish CO, an integral part of the Introduction program, was streamlined in 2010 by a national law [23]. It is delivered as a course, with a minimum duration of 60 h with learning outcomes related to four knowledge areas, divided into eight learning modules (shown in Fig. 1). The provision of CO is the responsibility of the municipalities that cooperate and coordinate regionally [23, 24]. The course is commonly provided in the largest native languages of participants and delivered by native speaking teachers, referred to as civics communicators. Health information, e.g. the right to health care and how to promote and take care of one’s health in Sweden is included in the course. Based on observed needs, some regions and municipalities offer additional hours of health communication [25]. The added health content includes topics that cover (1) the health care system, (2) self-care, i.e. how to take care of and improve one’s health through life-style changes and (3) other topics of relevance for migrants such as migration and health and mental health, family structure and sexual health. Health communication, defined here as health information delivered in the form of dialogue with participants, is seen by the stakeholders as enabling participants to improve their health, which in turn would allow them to benefit more from introduction activities [24]. The dialogue element was initially discussed and adopted in the bylaw of the CO ordinance [23] to encourage dialogue and reflection as opposed to one-way communication. However, few published studies have examined the potential of the CO with its embedded health communication, e.g. implementation and outcomes, especially from the point of view of the participants [26]. Previous Swedish studies include perceptions of sexual health information [27], health status and self-reported quality of life [28], impact of health information on health outcomes [29], and organisational and collaborative aspects in relation to introduction activities from the stakeholders’ point of view [30, 31]. Research from European and OECD countries on integration policies including CO focus on theoretical conceptualisations and governance aspects [32, 33] and economic, social, and political integration outcomes [34–36]; however, the health promotion perspective is generally lacking.

### Theoretical framework

*Social determinants of health* (SDH) refer to the relationship between an individual’s health, and the surrounding social and community networks, living and working conditions, and general socioeconomic, cultural and environmental factors [37]. The study was guided by the concept of SDH throughout, based on the aim of the CO being closely linked to SDH, i.e. to reduce inequalities faced by new migrants by facilitating establishment on the labour market and in society. SDH includes a power dimension and has been defined as ‘life conditions shaped by the distribution of money, power and resources at global, national and local levels’ [38]. Migration involves challenging processes beyond individual behavioural and cultural adaptations to a new context; it encompasses a complex and lengthy process of interactions and negotiations with new social, political, and economical structures [39], a process that intercepts our study. *Empowerment* is a concept linked to SDH, focusing on processes that moderate social inequalities [40]. Empowerment theory connects individual well-being with the larger social and political environment and has been defined as the process of enabling people to increase their control or power in all ways possible, through resources, education and awareness and by which one can achieve goals and participate in society [40]. We will employ empowerment theory to discuss findings in this study from the bottom-up perspective i.e. that of the participants.

The aim of the study was to explore participants’ perceptions and experiences of Swedish civic orientation with added health communication.

## Methods

### Study design and setting

An explorative qualitative design was used, a study design commonly employed to explore less studied phenomena [41]. Data were gathered through focus group discussions (FGD), a method used to attain a variety of perspectives and increase confidence in patterns that emerge [41]. Focus groups provide a social context and allows for both the collective and the individual to emerge; it enables participants to voice their views in the context of the views of others [42].

The study was carried out in Stockholm county. We had access to participants through the *Centre for Civic Orientation in Stockholm County*, a coordination office for 26 municipalities. About 190 courses were delivered in multiple schools and other locations across the municipalities during the study period. The CO was provided in the ten most prevalent languages used by participants at the time, with Arabic accounting for a large majority of the courses [43]. Three different schools in two municipalities were included for recruitment purposes.

Each CO-course started with 12 h of health communication delivered by external, native speaking, professional health communicators employed by the regions, followed by the regular CO of 60 h. An overview of the course content is shown in Fig. 1.

### Recruitment and participants

The study participants were purposively recruited from CO classes in the three selected schools. The inclusion criteria were to have completed the course and to speak one of the three languages in which the FGDs were conducted: Arabic, Somali and Farsi. The data collection was performed during the period June to August 2015.

Native speaking personnel associated with the research team visited the classrooms and informed participants about the study orally and handed out written information 1 day in advance of the FGD. The information included the purpose of the study, confidentiality in handling the data and how it would be stored and presented. Furthermore, that the FGD would be recorded for the purpose of transcription and that participants could discontinue participation at any time. Those who agreed to participate gave an oral consent and they were then invited to the FGD that took place at the schools, after class-hours on the last day of the course. Consent was thus given orally by the participants ahead of their participation in the FGDs and subsequently confirmed by their participation.

A total of 32 individuals participated in six focus groups. Two were held in Arabic, two Farsi and two in Somali, with 4–6 participants in each focus group. A majority had received their residence permit the same year as the study. All groups were mixed male and female participants, in the ages of 20–65 (see Table 1). It is recommended to have heterogeneity in the composition of the groups, which was sought to ensure variation in experiences and views based on e.g. gender, age and educational backgrounds [44]. Concurrently, the shared language, migration experience and having participated in the CO course gave a familiarity that enabled the participants to discuss freely.

**Table 1:** General characteristics of study participants (*N* = 32)

Factor	Number (n)
**Gender**	
Women	16
Men	16
**Age**	
20–30 yrs	9
31–40 yrs	10
41–50 yrs	7
51–65 yrs	6
**Native language**	
Arabic	12
Farsi	11
Somali	9
**Educational level**	
None	7
1–6 yrs	3
7–9 yrs	7
10–12 yrs	9
More than 12 yrs	6
**Year between receiving residence permit and CO course**	
Less than 1 year	20
1–2 years	11

### Data collection

An interview guide with semi-structured questions comprising four main questions and probing questions was developed. The guide was pilot tested through peer-review by one researcher working with qualitative methodology outside the research team, and one native-speaking moderator with cultural competence and experience of leading FGDs. The guide was translated into the three languages, and back translated to ensure quality and accuracy [45].

All FGDs were moderated by native speaking moderators with cultural competence [46] and relevant academic backgrounds. The same moderator conducted the FGDs for each language. First author moderated the Arabic speaking FGDs and participated in the other FGDs as an observer and was responsible for practical arrangements [42]. Interaction between the participants e.g. how much participants spoke, body language, etc. was observed and noted. Short debriefing sessions was held with the Somali and Farsi moderators following each FGD to confirm and compare overall impressions of the interactions. The FGDs lasted 45–60 min. All FGDs were recorded and simultaneously translated and transcribed into Swedish by the moderators, except for the Somali ones, which were translated and transcribed by an external person.

At the time of the study, the research team consisted of five researchers: one male and one female professor and one female associate professor, responsible for the conceptualisation of the study; and one female post-doc and one female PhD student, responsible for project administration and data collection. The research group have broad experiences in research and practice involving refugees and migrants, including health promotion and intervention programs, health literacy, sexual and reproductive health and rights, as well as qualitative methodology. Three of the four authors are Swedish-born and one, the first author, has an immigrant background with cross-cultural and linguistics skills relevant for the project [46]. Ethical approval for the study was applied for, and granted by the Regional Ethical Review Board of Uppsala, Sweden (registration number 2014/526). Ethical considerations in the study included informing participants comprehensively, both orally and in writing in their native language about the study and FGD process. This was done to ensure that the consent to participate was based on information about what participation would entail. We stressed the importance of keeping the information that was shared in the FGD within the group, as confidentiality otherwise cannot be ensured in a FGD setting [41]. The information also included possibility to discontinue participation and confidentiality in handling, storing and presenting the data.

### Analysis

The data were analysed following the steps described by Krueger and Casey [42], which is a method used for content analysis of focus group discussions. It stipulates a distinction between descriptions and interpretations of the data. When using focus groups, it is encouraged to capture group interaction between participants as well as the display of such interaction in the presentation of the data [42].

The analysis started during the data collection with the first author reading the transcripts as they were finalised for each FGD and writing short summaries. After the completion of the data collection, the transcripts were read again several times and sorted roughly based on the questions in the interview guide as a way to identify patterns [42]. The transcripts were coded based on those patterns, and similar codes were merged together and labelled with sub-categories. The coding was performed in several rounds by the first author, in an iterative, comparative process going back and forth between transcripts, quotations and codes. The other authors then read the transcripts and coded parts of them. The codes and sub-categories were compared, discussed and adjusted in a process involving all authors. After a final round of discussions, sub-categories were merged to form three main categories.

The analysis was performed in Swedish from the Swedish transcripts to avoid interpretation due to translation occurring in the analysis. Sub-categories, categories and quotations were translated into English once the analysis was completed.

Investigator triangulation was performed to validate findings; sub-categories and final categories were reached jointly, after extensive work and discussions. Quotations were used to illuminate main findings and interaction between participants. Reflexivity was considered throughout the analysis, in terms of possible biases created by pre-conceived ideas based on differences between participants and the researchers in social status, education and background, and countered by the analytical triangulation that was performed [41].

## Results

Three main categories were identified: (1) *The course gives* v*aluable information but needs adjustments, (2) The health communication inspired participants to focus on their health, (3) Participation in the course promoted independence and self-confidence.* Each category is based on two sub-categories described below.

### The course gives valuable information but needs adjustments

This category is composed of the sub-categories: (1) *civic and health orientation needed earlier – during the asylum phase* and (2) *planning and course content need adjustments.*

A main finding was that the course provides information and guidance about the Swedish society and health; information that the participants felt they would have needed earlier in their resettlement process. Information about the healthcare system, the laws, education and the labour market were specifically mentioned as valuable and essential for starting a life in Sweden. Some participants reasoned that the course could give meaningful activity and improve well-being during the lengthy asylum phase, otherwise marked by waiting.

*- This course is like a compass to us. So, if I was responsible for the course, I would let every newly arrived refugee take it directly after arrival, regardless of if they receive the permit or not. To give this compass that will guide the way to wherever he or she wants to go, to ideas … through work or studies or anything else. A compass that starts early, so that it can guide the people. (FGD # 1)*

Another reason why it would be important to get the course early was that it gives accurate information from a competent authority. This, in turn, would help them avoid misinformation, resulting in involuntary mistakes. Lack of information made the participants confused and dependant on hearsay.

*- During this year (waiting for decision), you hear a lot of things and claims. So, you get faulty information and you also get some correct information. It’s important that you get the information in an early stage, so that you understand the laws in the right way, and it becomes established in a permanent way. Then, you can avoid the uncertainty; you get the right information directly. (FGD # 1)*

Participants mentioned that the teachers were competent and committed which created a good atmosphere and facilitated discussions about different topics. The teachers’ communication in the participants’ language was experienced as being a mediator and enabling factor. Another finding related to the course design was that more time is needed for the course, to allow for more discussions and elaborations. Many participants expressed that the course was too compressed and some parts too detailed, which was perceived as challenging, especially given that the course organisation did not provide any written information. There was also a preference across all focus groups to have written course material, in order not to miss out or forget about important information given in class. They also mentioned that the health topics should be introduced later in the course to facilitate learning and discussion about topics which they perceived as being sensitive, like sexual health and relations. Moreover, adjusting the course content to the participants’ age and previous knowledge was viewed as important. This was discussed in relation to topics that were less context bound, e.g. health topics, where some participants had previous knowledge while others did not. The heterogeneity of the group was seen by some as a positive feature, but more recurrently a challenge:

*- The teacher explained about an app and how to count calories. It’s difficult for the older persons to use the mobile and understand this. But for my age, it’s really easy. I understand it directly, but they need a long time to understand such a thing; they haven’t really been exposed to it earlier. (FGD # 2)*

### Health communication inspired participants to focus on their health

This category is composed of the sub-categories: (1) *Useful information about health and healthcare* and (2) *Health communication inspired healthier habits*.

Participants experienced the health topics as providing important information, regarding the right to healthcare, patient fees, children’s health, women’s health, mental health and well-being, pharmacies and the right to have an interpreter. They viewed the information about how to seek regular and emergency healthcare as particularly important. Many participants felt they had acquired an understanding of how and when to seek healthcare.

*- For me, it has become much easier than before the course. If I get sick today, I can call 1177 with confidence. Or, if I’m not too sick I can call the healthcare centre and book an appointment with a doctor. Now I understand how the healthcare works: if I call and get the advice to stay at home, then I understand that there are others who are in more acute need of healthcare. I didn’t understand this before and used to get frustrated when I didn’t get an appointment at the doctor’s. (FGD # 3)*

The health topic of sexual health and relations was discussed among the participants in some of the focus groups. The common ground was that information about sexual health was necessary and much needed due to the lack of information and difference in values between Sweden and their countries of origin. Some had pre-existing knowledge about the topic, but several participants were of the view that the information about sexual health was unavailable to them in their countries of birth, as shown in this interaction:

*- We come from a country where we don’t talk about sexual relations [ … ] What I mean is that most of us don’t know much about sexuality and sexual relation; we are ashamed to talk about it. For example, about contraceptives, how to avoid an unwanted pregnancy and how to protect yourself from sexually transmitted diseases. (Participant #03, FGD 3)*

*- I agree with you, in =Iran= we never spoke about sexual relations with an unmarried person. It was expected of you to know everything when you were married but there was no information. It wasn’t until I got here that I found out about sex and relationships. (Participant # 06, FGD 3)*

*- I don’t think that =Iran= differs from here. Just like here, there are people there that have more or less information about sexual health and relations. It’s not like everyone in =Iran= has the same information. (Participant #04, FGD 3)*

The topics regarding lifestyle such as healthy eating habits, daily exercise and how to change health habits evoked a lot of interest from the participants. They perceived this information as detailed and trustworthy. Many participants expressed having been inspired to take up new health habits like keeping good sleeping habits, eating healthier foods, and doing exercise on a daily basis.

*- About health, I would like to add that to take care of one’s health is a start of our integration in Sweden. (FGD # 1)*

For some, most of the general information on healthy choices was not new, however context bound information was, such as specific labelling of healthy food choices in stores, organic fruit and low-fat products. The health communication worked as a motivation to activation, being outdoors with children more frequently; avoid taking the bus and walking longer distances. The health communication in the civics orientation was expressed as something positive and inspiring. Taking care of one’s health was perceived as an area given much importance by authorities and people in general in Sweden and therefore pertinent to them as citizens to be.

*- Another thing she (the teacher) talked about was the equality in exercising; it’s not only the men who should have strong physique, but women can also contribute to society with their muscular strength, in addition to their intellectual capacity of course. (FGD # 1)*

### Participation in the course promoted independence and self-confidence

This category is composed of the sub-categories: (1) *The course gives insights into society and values in Sweden* and (2) *Participation in the course gives independence and new social contacts.*

The information about how the Swedish society is organised through laws and institutions was as eye-opening experiences for the participants. Through the information about family relations, children’s education, the individual’s liberties and rights and social codes, participants expressed having gained a clearer understanding and awareness of aspects of society that were ambiguous before.

*- It (the course) is very good for us. When you come here, you are like a blind person, even if you can read and write. Without information about the society, you have to feel your way around all the time, but with a course like this you get the knowledge you need to function in society. (FGD # 4)*

Many participants experienced the new-gained awareness and knowledge as a pathway to self-confidence in encounters with societal institutions. Children’s needs, health and school attendance are areas that were viewed as having become easier to navigate.

*- We can get equal healthcare, and care everywhere. No one can say that we can’t get healthcare because we are new in the country. This I have learnt, and I have become self-confident and know my rights in the healthcare system. (FGD # 5)*

Even when discussing sensitive topics, such as family relations and laws on marriage, participants experienced a positive impact of knowing society’s views and comparing it with their own*.* Some participants perceived the information as giving them hope and goal orientation for future studies and work, something which they expressed had affected them on a deeper level.

*- I have learnt that I can have an influence in society and have my own personality, be myself. And how to study and work to improve your self-confidence; that I have learnt. (FGD # 1)*

Across the focus groups, the participants expressed that they interacted significantly with the teachers who also inspired participants to learn the language and partake in society as they had done. An experience that was shared by many participants was that the course had activated them socially and provided them with opportunities to make friends and meet people from other provinces and countries. Participants also reported having reached out to others in their communities to assist or guide in matters that they had gained knowledge about, such as how to get access to healthcare.

## Discussion

This study explored newly settled migrants’ perceptions and experiences of Swedish civic orientation and health communication. It adds new knowledge on the users’ perspectives on the potential of CO to promote health and integration. The findings suggest that CO offers valuable guidance, inspired participants to think about their health and increased their self-confidence and independence, i.e. was empowering. However, in order to increase the health promotion potential of the course, it needs adjustments to better fit the participants’ circumstances and pre-existing knowledge. Studies involving the end users’ perspectives on introduction activities are sparse but important, as they have relevance for policy and significance for stakeholders to meet their aims.

The participants expressed that the information they received in the CO made their contacts with authorities, e.g. healthcare system, easier to navigate and more accessible to them. In addition, the course gave new insights and understanding about how the Swedish society is organised. These findings are consistent with the *outcomes* of the CO being empowering, as it gave situation-specific perceived control and skills [40]. Comparable findings regarding increased self-confidence and autonomy as an outcome of civic and health information has been reported in studies on immigrant parent education programs [47] and culturally tailored health programs [48]. Moreover, the study found that CO provided opportunities to develop knowledge and skills to gain more control over one’s life and participate in the Swedish society, as well as gaining new social contacts. This is consistent with the *process* itself, i.e. participation in CO, being empowering and not only the outcomes themselves [40]. It can be theorised that the social interaction which occurred between the participants as well as between the participants and the teachers is a form of bonding and bridging social capital [49], i.e. trust and sharing within a homogenous group, and engagement between individuals who are different in terms of social power. Developing social networks and social participation can strengthen resilience against poor mental health among newly settled migrants [16].

Yet, if participant’s perspectives were to be taken into account on how the course could be adjusted to better fit their needs, the process could be made more empowering. A critical discourse analysis of governmental policy documents on migrants in the Nordic countries showed that the empowering perspective was largely lacking in the analysed documents, placing migrants in a passive position vis-à-vis decision-making [50]. Another aspect is that health promotion in introduction activities requires more intersectional collaboration and a clearer definition of roles between involved authorities in order to be fully realised e.g. the municipalities, the Public Employment Services and the regions in charge of healthcare [24, 31]. The finding that CO is needed in the early asylum phase relates to the phase being marked by resettlement stressors such as waiting on residency status and family reunification, isolation, and low access to information [2, 8]; furthermore, it is an example of how intersectional collaboration is required as this phase is formally outside of the Introduction program. The need for context specific health information during the early asylum phase has been reported in other studies [51, 52]. In 2020, the Swedish government mandated a short compulsory CO course to be given to refugees during the asylum phase and increased the regular CO to 100 h [53], which corresponds to some of the needs expressed in this study, e.g. the course was compressed which was perceived as challenging. However, it is not evident that the need to adapt the delivery and content of CO to participant’s circumstances and pre-existing knowledge e.g. health literacy, has been adequately addressed in the new directive. The importance of adapting health information to health literacy levels and educational backgrounds of newly settled migrants has been echoed by other studies involving newly settled migrants in Sweden [17, 54]. Our study shows that the native speaking teachers acted as cultural mediators, enabling discussion about different topics. Earlier studies have found that cultural mediation not only decreases language and cultural communication barriers [55] but also mediates cultural differences and facilitates communication and knowledge about a given topic [56]. Intercultural mediation is used in Europe and OECD countries within the healthcare system [56], but less so in civics orientation [57], a practice that is supported by the findings of this study.

The added health communication proved to be an important component as it gave the participants useful information about health and healthcare and inspired healthier lifestyle habits. This is in line with studies on recently settled Arabic and Somali speaking women that found that short, tailored, health promotion interventions carried out by licensed health professionals improved participants’ self-rated health, empowerment and uptake of healthier habits [48]. Moreover, the information about sexual health was perceived as important and needed despite differences in values between home countries and Sweden, a finding in line with other studies involving migrants and access to sexual health information [27, 58]. However, an intervention study on resettled Iraqi migrants in Sweden and the effects of health communication by professional healthcare advisors found no association between self-reported knowledge on lifestyle factors and improved health status [29]. An explanation is that SDH factors such as prospects of long-term housing and work opportunities influence the buffering effect of health promotion in the resettlement process [39]. Nevertheless, similar to the information about society, the health communication increased the participants’ understanding and confidence in how to access and navigate the healthcare i.e. increased health literacy, which is linked to empowerment [15].

### Methodological considerations

Most of the study participants had permanent residence permits, as it was the most common form in Sweden at the time of the study. If we were to carry out the study today, with temporary residence permits being the standard, some of the results might be different since temporary residence permits are associated with more stress and anxiety [59]. All persons in the visited classes were invited to participate in the FGDs. It cannot be ruled out that those who were very critical opted not to participate. However, this is not evident to be the case since the results include both positive and negative perspectives about the CO course.

A limitation of the study is the mixing of male and female participants in the FGDs, which could potentially have hindered the female participants from making their voices heard, referred to as the peacock effect [42]. However, this was not evident in the observations of the interactions and may have been countered by the fact that the focus groups mirrored the earlier mixed classroom composition and the familiarity that the participants had with each other. A strength was the diverse composition of the focus groups, reflecting different perspectives of migrant and language groups in Sweden, including different ages and educational backgrounds within the respective groups [44].

Another limitation was that no observer was present in the Arabic speaking FGDs, due to logistical reasons. On the other hand, the first author moderated these FGDs herself. A strength of the study was that moderation of all FGDs was done in the participants’ native languages allowing for direct communication. This was by choice, as not using interpreters as middle hand allows for rich communication and openness [60]. Also, the translation and transcription of all but the Somali focus groups were done by the first author and the other moderators, which lowered the risk of misinterpreting the context or information being lost in the transcription process [60]. Shared language and culture between the moderators and the participants can create bias, e.g. certain meanings are taken for granted or sensitive topics avoided. However, this was not evident in the transcripts, likely mediated by the fact that all the moderators were trained and well established in the Swedish context.

## Conclusion

This study adds knowledge about the users’ perspectives on the potential of civic orientation to promote health and integration of newly settled migrants, describing ways in which civic orientation with added health communication promoted health and empowerment. The CO with added health communication provided valuable information about health and society, which inspired participants to focus on their health and promoted independence and self-confidence. However, the content and delivery of the course need adjustment to better fit migrants’ life situations and varying levels of pre-existing knowledge to increase their opportunities to benefit and engage actively in their establishment and integration process.

Based on the experiences of the participants, the potential to benefit from the CO course could be augmented if the following points were addressed by stakeholders:

- CO should be offered earlier in the resettlement process, especially essential aspects of it e.g. information about health and the healthcare system, laws and the labour market.
- More time should be allowed for the CO course, without infringing on time allotted for other activities in the Introduction program.
- The educational backgrounds of participants should be taken into account when forming CO groups.

## Supplementary Information

**Additional file 1.** Interview guide for FGDs.

## Data Availability

The data analysed during the current study are available from the corresponding author on reasonable request. To protect the participants’ identities, the full interview data of this study (transcripts and audio files) will not be made available to the public.

## Abbreviations

WHO: World Health Organisation
CO: Civic Orientation
SDH: Social Determinants of Health
FGD: Focus Group Discussion
