# Supplementary material for: “This course is like a compass to us” – a qualitative study on newly settled migrants’ perceptions of civic and health orientation in Sweden
Source: BMC Public Health. 2021 Aug 31;21:1606. doi: 10.1186/s12889-021-11654-3 (PMC8408945; doi:10.1186/s12889-021-11654-3)
Supplement: Supplementary file 1 — Additional file 1. Interview guide for FGDs. [file 12889_2021_11654_MOESM1_ESM.docx]

**Interview guide for FGDs**

**Opening question**

1. What do you think about the civic orientation for newly arrived migrants?

**Civic and heath information**

1. What do you think about the information that you received?
   - What type of information did you receive?
   - In what ways have you used the information/knowledge (if any)?
   - In what ways have you been affected by the information (if any)?

- Has the information been beneficial for you? If yes, in what ways?

**The health communication**

1. What do you think about the health communication within the civic orientation?
   - How did you perceive the content of the health communication?
   - How did you perceive the arrangement of the health communication?
   - How did you perceive the health communicators?
   - What were your experiences’ around understanding and discussing with others in the group, the topics that the health communicator brought up?

**Concluding question**

1. If you were at liberty to change anything, how could the civic and health orientation be delivered to be more beneficial to you and your health?
